# Employment status, productivity loss, and associated factors among people with multiple sclerosis

**DOI:** 10.1177/13524585231164295

**Published:** 2023-04-15

**Authors:** Elisabet Rodriguez Llorian, Wei Zhang, Amir Khakban, Kristina Michaux, Scott Patten, Anthony Traboulsee, Jiwon Oh, Shannon Kolind, Alexandre Prat, Roger Tam, Larry D Lynd

**Affiliations:** Collaboration for Outcomes Research and Evaluation (CORE), Faculty of Pharmaceutical Sciences, The University of British Columbia, Vancouver, BC, Canada; School of Population and Public Health, The University of British Columbia, Vancouver, BC, Canada Centre for Health Evaluation and Outcome Sciences (CHÉOS), St. Paul’s Hospital, Vancouver, BC, Canada; Collaboration for Outcomes Research and Evaluation (CORE), Faculty of Pharmaceutical Sciences, The University of British Columbia, Vancouver, BC, Canada; Collaboration for Outcomes Research and Evaluation (CORE), Faculty of Pharmaceutical Sciences, The University of British Columbia, Vancouver, BC, Canada; Department of Psychiatry, University of Calgary, Calgary, AB, Canada; Division of Neurology, Department of Medicine, The University of British Columbia, Vancouver, BC, Canada; Division of Neurology, St. Michael’s Hospital, University of Toronto, Toronto, ON, Canada; Division of Neurology, Department of Medicine, The University of British Columbia, Vancouver, BC, Canada; Department of Neurology, Faculty of Medicine, Université de Montreal, Montreal, QC, Canada; School of Biomedical Engineering, The University of British Columbia, Vancouver, BC, Canada; Collaboration for Outcomes Research and Evaluation (CORE), Faculty of Pharmaceutical Sciences, The University of British Columbia, Vancouver, BC, Canada Centre for Health Evaluation and Outcome Sciences (CHÉOS), St. Paul’s Hospital, Vancouver, BC, Canada

**Keywords:** Employment status, productivity loss, multiple sclerosis

## Abstract

**Background:**

Multiple Sclerosis (MS) affects people in their most productive years of life. Consequently, MS can substantially affect employment and work-related outcomes.

**Objectives:**

This study characterizes productivity loss and employment status of people with multiple sclerosis (pwMS) and investigates associated factors.

**Methods:**

We used baseline data collected as part of the Canadian Prospective Cohort Study to Understand Progression in Multiple Sclerosis (CanProCo). Using the Valuation of Lost Productivity questionnaire, we measured MS-related paid work productivity loss for those employed, productivity losses incurred by those unemployed (i.e. lost employment time), and unpaid work productivity losses for all. A set of sociodemographic, disease, and performance-related factors were investigated using a two-part regression model for productivity loss and a multinomial logistic model for employment status.

**Results:**

From the cohort of 888 pwMS enrolled at baseline (mostly showing mild to moderate disability), 75% were employed, and of those unemployed, 69% attributed their unemployment to health-related issues. Total productivity loss over a 3-month period averaged 64 and 395 hours for those employed and unemployed, respectively. Some factors that affected productivity loss and employment status included use of disease-modifying therapies, fatigue, and performance indicators such as cognitive processing speed.

**Conclusion:**

Productivity loss experienced by employed and unemployed pwMS is substantial. Targeting the identified modifiable factors is likely to improve work productivity and permanence of MS patients in the workforce.

## Introduction

Diagnosis for multiple sclerosis (MS) typically occurs in early adulthood.^1^ Consequently, MS affects work activity and occupational outcomes.^2^ People with multiple sclerosis (pwMS) experience substantial productivity losses and associated costs,^3,4^ which can take many forms: pwMS may completely exit the workforce or remain employed but frequently miss time from work (absenteeism) or exhibit reduced work productivity while at work (presenteeism). In addition, regardless of employment status, pwMS also experience productivity losses from unpaid activities such as housework, shopping, or childcare.

A study in Sweden estimated that after a 10-year follow-up, only 28% of pwMS were working full-time and 23% part-time.^5^ Even though there is some variability across countries and, in the last years, the proportion of unemployed pwMS has decreased (potentially related to advances in disease-modifying therapy (DMT)), pooled estimates for unemployment and early retirement remain 35.6% and 17.2%, respectively.^6^ Furthermore, many pwMS that are still working experience substantial productivity losses even at low severity levels, as reported in our previous study in Canada, which showed an estimated 60 hours of productivity lost among employed pwMS during a 3-month period, including, presenteeism, absenteeism, and unpaid work loss.^7^

The relationship among demographic, clinical, physical, and cognitive impairment factors and employment and work-related outcomes (including employment status, working hours, and productivity loss) have been studied previously.^8,9^ However, with a few exceptions,^10–12^ a comprehensive set of factors have not been investigated conjointly and compared across several employment and work-related outcomes. Understanding the factors that may contribute to workforce retention for pwMS, specifically identifying those that are modifiable, is highly relevant when providing guidance for policy design and treatment that can improve work productivity, health outcomes, and quality of life (QoL) of pwMS.

The objective of this study is to provide a comprehensive assessment of employment status and productivity losses in a Canadian cohort of pwMS with early disease severity and identify specific factors that are associated with employment status and productivity loss.

## Materials and methods

### Data and cohort

This study uses information collected at baseline from the Canadian Prospective Cohort Study to Understand Progression in Multiple Sclerosis (CanProCo), a 5-year prospective cohort study conducted in five sites across four Canadian provinces (Alberta, British Columbia, Ontario, and Quebec) with the primary aim to better understand MS disease progression.^13^ Details on CanProCo inclusion criteria, ethics, and informed consent are provided in the Supplemental Appendix (SA). The CanProCo baseline cohort includes only people of working age (19–64 years old).

### Productivity loss outcomes and associated factors

There are two primary outcomes of interest: (1) productivity loss (measured as a continuous variable in hours) and (2) employment status, a categorical variable with four groups: (a) employed full-time, (b) employed part-time, (c) unemployed due to health reasons, and (d) unemployed due to other non-health reasons. Self-employed pwMS were categorized as full- or part-time employees based on the number of days they worked per week. Both outcomes are collected using the Valuation of Lost Productivity (VOLP) questionnaire, which collects self-reported productivity in the 3 months prior to the collection date, and has been previously validated and applied in other diseases.^14,15^ Productivity loss includes paid work productivity loss (i.e. both absenteeism and presenteeism) because of health problems for those employed, productivity loss incurred by pwMS under 65 years who are unemployed because of health problems (i.e. lost employment time), and unpaid work productivity losses for all patients. The SA provides additional details on measuring paid work loss (presenteeism and absenteeism) and unpaid work loss derived from unpaid activities which can include housework, shopping, and childcare, among other. For calculating lost employment time among unemployed pwMS, we use Statistics Canada^16^ average working hours per week for both full-time and part-time employment, multiplied by 13 weeks for 3 months. Average working hours do not vary substantially across Canadian provinces.^16^

Based on Rodriguez Llorian et al.,^7^ and information collected as part of CanProCo,^13^ a set of sociodemographic, disease, and performance-related indicators were selected to investigate their associations with productivity loss and employment status. Sociodemographic variables included sex and age. Disease indicators included time since diagnosis in years; severity of disease measured using the Expanded Disability Status Scale (EDSS), which ranges from 0 to 10, with 0 representing no disability; fatigue using the Modified Fatigue Impact Scale (MFIS)^17^ ranging from 0 to 84, with higher scores signaling higher levels of fatigue; DMT use; number of comorbidities; whether the patient had a relapse in the past 3 months; and MS phenotype. Health-related QoL utility was measured using the EQ-5D-5L instrument^18^ and associated value set for Canada.^19^ Finally, performance indicators included cognitive processing speed, visual acuity, manual dexterity, and walking speed; details of which can be found in the published protocol of the CanProCo study.^13^ Cognitive processing speed and visual acuity are measured in terms of number of correct responses in the corresponding test, while manual dexterity and walking speed are measured in time (seconds) needed to complete a specific task. Consequently, higher values of the former measures signal higher performance, while the latter timed scores represent a deterioration in health status.

### Statistical analysis

Two different types of models were used to measure the association between the pre-selected factors and study outcomes. For estimating productivity loss, due to the highly skewed nature of the data, we used a two-part regression model. The model first conducts a logistic regression to estimate the probability of having zero productivity loss, followed by a generalized linear model (GLM) with a log link and gamma distribution, fitted for those participants showing non-zero productivity loss—combined, these models generate a marginal (or incremental) effect of each factor on productivity loss.^20^ For the employment status outcome, a multinomial logistic regression was conducted, with the regression coefficients translated into odds ratios. For both models, only those variables with a *p*-value ⩽ 0.1 in the univariate models coefficients (joint coefficients for the two-part models)^20^ were included in the final multivariate analysis.

## Results

### Cohort characteristics

**Figure 1.**
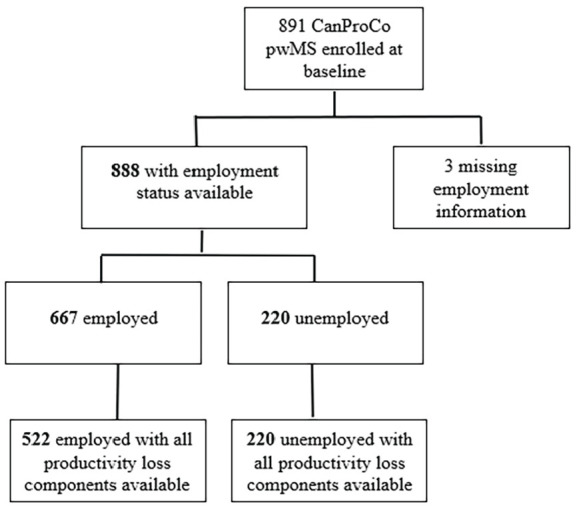
Study cohort.

The participant flow chart of the initial 891 CanProCo patients enrolled at baseline is presented in Figure 1. Discarding three patients with missing employment information, of the 888 patients with reported employment status, 667 (75%) were employed and 221 (25%) were unemployed.

Table 1 presents the characteristics of the 888 pwMS separated by their employment status. Overall, the cohort of participants were mostly female (70%), with a relapsing-remitting MS-type (79%) and mild to moderate disability (71% with EDSS 1–3.5). The mean age of the cohort was 40 (standard deviation (SD) = 10.3) years, and disease duration was 3 (SD = 2.7) years. Unemployed pwMS were older, had more severe MS, experienced a recent relapse, were more likely to have a primary-progressive MS, or had more than three comorbidities compared to employed pwMS (all *p* < 0.1). Likewise, employed participants reported having less fatigue, better QoL, and performed better on the cognitive processing speed, manual dexterity, and walking speed tests.

**Table 1.** Characteristics of pwMS.

Variable	Employed pwMS (*n* = 667)		Unemployed pwMS (*n* = 221)		*p*-value^a^
	*N* ^b^	Statistic	*N*	Statistic	
Sex, % female	667	467 (70%)	221	158 (72%)	0.68
Age (years), mean (SD)	667	39.0 (9.6)	221	40.7 (12.1)	0.03
Severity^c^					<0.01
No disability EDSS 0	666	161 (24%)	221	27 (12%)	
Mild disability EDSS 1–3.5	666	482 (72%)	221	150 (68%)	
Moderate disability EDSS 4–6.5	666	23 (4%)	221	44 (20%)	
EDSS, median	666	1.5	221	2.5	
Time since diagnosis (years), mean (SD)	667	3.2 (2.7)	221	3.3 (3.0)	0.44
MS type, % by phenotype					
RRMS	667	529 (78%)	221	171 (77%)	0.54
PPMS	667	44 (7%)	221	33 (15%)	<0.01
RIS	667	51 (8%)	221	11 (5%)	0.18
CIS	667	43 (7%)	221	6 (3%)	Ref.
Current DMT users, %	667	342 (51%)	221	120 (54%)	0.44
Relapsed in the past 3 months, %	640	48 (8%)	211	24 (11%)	0.08
Comorbidities, %					
0	667	253 (38%)	221	51 (23%)	Ref.
1	667	173 (26%)	221	61 (28%)	0.62
2	667	118 (18%)	221	44 (20%)	0.46
+3	667	123 (18%)	221	65 (29%)	<0.01
Fatigue^d^, mean (SD)	641	26.9 (18.2)	208	44.7 (20.9)	<0.01
EQ-5D utility score, mean (SD)	663	0.9 (0.1)	215	0.8 (0.1)	<0.01
MS performance test^e^, mean (SD)					
Cognitive processing speed	661	57.1 (10.6)	217	49.4 (12.6)	<0.01
Visual acuity	615	58.6 (5.2)	191	57.9 (4.6)	0.12
Manual dexterity	657	22.9 (5.9)	215	28.1 (16.4)	<0.01
Walking speed	657	4.9 (1.4)	216	6.8 (5.0)	<0.01

EDSS: Expanded Disability Status Scale; RRMS: relapsing-remitting multiple sclerosis; PPMS: primary-progressive multiple sclerosis; RIS: radiologically isolated syndrome; CIS: clinical-isolated syndrome; DMT: disease-modifying therapy; SD: standard deviation; pwMS: people with multiple sclerosis; Ref.: reference category.

a Employed and unemployed participants were compared using a *t*-test for continuous variables described using a mean and standard deviation, and a chi-square test for categorical variables.

b Respondents with non-missing information included in the analysis of each variable.

c EDSS is measured as a continuous variable in the statistical analysis.

d Measured using MFIS, score ranging from 0 to 84.

e Measured using MSPT: for processing speed measured as number of correct responses out of 110, visual acuity as number of correct responses out of 60; manual dexterity and walking speed as time taken to complete specific tasks in seconds.

### Productivity loss and employment status

Employed pwMS worked an average of 36.7 (SD = 11.6) hours per week, with 71% working full-time, 15% part-time, and 14% self-employed (Table 2). Among the unemployed, 69% reported being unemployed because of health reasons, which included those on work disability and various other reasons for not working such as early retirement.

**Table 2.** Employment status, working hours, and productivity loss.

	*N*	Statistic
**Employed pwMS**		
Working full-time	667	472 (71%)
Working part-time	667	102 (15%)
Self-employed	667	93 (14%)
Hours worked per week, mean (SD)^a^	580	36.7 (11.6)
Days worked per week, mean (SD)^a^	656	4.8 (1.0)
Productivity loss hours amongst employed, mean (SD)	522	63.6 (107.7)
Paid work productivity loss—absenteeism	522	19.9 (49.9)
Paid work productivity loss—presenteeism	522	25.5 (56.6)
Unpaid work productivity loss	522	18.2 (58.3)
**Not employed pwMS**		
On official work disability	221	91 (41%)
Unemployed but looking for work	221	29 (13%)
Unemployed but not looking for work	221	24 (11%)
Unemployed with no other information	221	15 (7%)
Retired	221	22 (10%)
Housewife/househusband	221	22 (10%)
Student	221	18 (8%)
Unemployed due to health, *N* (% of total unemployed pwMS)	221	152 (69%)
Productivity loss hours amongst unemployed, mean (SD)	220	395.4 (309.0)
Lost employment time^b^	220	335.0 (224.6)
Unpaid work productivity loss	220	60.4 (170.2)

SD: standard deviation; pwMS: people with multiple sclerosis.

a Calculated among employed pwMS.

b Only calculated if pwMS is unemployed due to health (these include all those on official work disability, as well as some on early retirement or unemployed due to health reasons). One person did not answer whether unemployment status was due to health reasons.

Approximately 62% of participants had some form of productivity loss (Table 2). For those employed, productivity loss averaged 63.6 (SD = 107.7) hours per person over a 3-month period, with the biggest proportion being presenteeism (41%), followed by absenteeism (31%), and unpaid productivity loss (28%). Assuming an 8-hour workday, 64 hours translate to approximately 2.5 days per month of lost productivity. For those unemployed, productivity losses averaged 335 hours per person over 3 months, which was mainly driven by the losses of those on work disability. Unemployed participants also incurred unpaid productivity losses, totaling 60.4 (SD = 170.2) hours over 3 months, which was higher than unpaid losses incurred by employed pwMS (18.2 hours).

### Factors associated with productivity loss and employment status

After conducting univariate analyses (SA), statistically significant variables were then evaluated for inclusion in a final multivariate model. Below we outlined results for selected variables found to be statistically significant, mainly sex and some modifiable factors with potential relevance for policy design including severity, DMT use, relapses, fatigue, and performance indicators. A complete set of results is presented in Table 3. The first two columns show results from the multivariate two-part models using total productivity loss as outcome, separated by employed (Model 1, Table 3) and unemployed pwMS (Model 2, Table 3). The remainder of the columns (Model 3) shows a multinomial logistic regression using employment status as the outcome and full-time employment as the base category.

**Table 3.** Factors associated with productivity loss and employment status indicators—multivariate.

	Model 1—productivity losses employed pwMS, marginal rates	Model 2—productivity losses unemployed pwMS, marginal rates	Model 3—employment status, odds ratios (full-time employment is base category)		
			Part-time	Unemployed due to health	Unemployed not due to health
Female	2.76 (−16.49, 22.00)	**43.01 (−25.44, 111.46)**	**2.17 (1.22, 3.87)**	1.41 (0.74, 2.66)	1.52 (0.77, 3.01)
Age (years)	**−0.36 (−1.41, 0.69)**	**3.50 (0.60, 6.40)**	**0.97 (0.94, 0.99)**	1.02 (0.99, 1.05)	**0.94 (0.91, 0.97)**
EDSS	**18.71 (7.07, 30.35)**	−6.98 (−32.39, 18.43)	1.04 (0.82, 1.33)	**1.32 (1.00, 1.74)**	**1.52 (1.10, 2.12)**
Time since diagnosis	**−2.78 (−6.40, 0.84)**	–	–	–	–
MS phenotype					
RRMS	−7.99 (−39.49, 23.50)	**−100.25 (−216.26, 15.76)**	0.97 (0.37, 2.59)	6.70 (0.65, 68.83)	1.56 (0.41, 5.96)
PPMS	**−**15.72 (−56.03, 24.59)	**−116.82 (−228.24, −5.41)**	0.41 (0.08, 2.11)	3.08 (0.26, 37.30)	1.44 (0.23, 8.92)
RIS	−18.67 (−54.67, 17.34)	**−191.72 (−316.32, −67.12)**	1.12 (0.34, 3.68)	2.84 (0.21, 39.45)	2.78 (0.57, 13.60)
CIS	Ref.	Ref.	Ref.	Ref.	Ref.
Current DMT use	9.66 (−10.11, 29.44)	**62.91 (−5.21, 131.03)**	1.06 (0.65, 1.72)	**1.82 (0.98, 3.36)**	**0.58 (0.30, 1.10)**
Relapse	**49.20 (−12.67, 111.08)**	–	**2.10 (0.99, 4.43)**	2.95 (1.14, 7.61)	1.73 (0.66, 4.59)
Comorbidities					
0	Ref.	Ref.	Ref.	Ref.	Ref.
1	−14.97 (−36.05, 6.12)	**−110.66 (−197.22, −24.11)**	1.00 (0.56, 1.80)	0.81 (0.36, 1.81)	**2.85 (1.43, 5.68)**
2	1.85 (−22.59, 26.30)	−59.24 (−159.45, 40.96)	**1.76 (0.95, 3.27)**	1.04 (0.44, 2.46)	1.33 (0.51, 3.51)
3+	**8.40 (−16.40, 33.20)**	−35.26 (−136.05, 65.53)	0.66 (0.31, 1.37)	0.84 (0.37, 1.92)	0.61 (0.19, 2.02)
Fatigue index MFIS	**2.29 (1.57, 3.01)**	**5.33 (3.02, 7.64)**	**1.01 (1.00, 1.03)**	**1.06 (1.04, 1.08)**	1.00 (0.98, 1.02)
EQ-5D utility score	11.90 (−0.79, 24.59)	**−36.69 (−78.87, 5.50)**	0.86 (0.66, 1.13)	**0.79 (0.61, 1.01)**	1.23 (0.80, 1.89)
Cognitive processing speed	–	**−2.13 (−4.92, 0.67)**	**0.95 (0.93, 0.97)**	**0.95 (0.92, 0.98)**	**0.95 (0.93, 0.98)**
Visual acuity	**−0.64 (−1.74, 0.46)**	−2.40 (−17.94, 13.14)	1.03 (0.96, 1.11)	1.04 (0.98, 1.09)	1.09 (0.94, 1.25)
Manual dexterity	–	–	0.96 (0.91, 1.02)	**1.03 (1.00, 1.07)**	1.03 (0.99, 1.07)
Walking speed	−2.81 (−11.77, 6.16)	−0.63 (−13.08, 11.84)	0.97 (0.81, 1.16)	1.04 (0.93, 1.17)	1.09 (0.94, 1.26)

Ref.: reference category; EDSS: Expanded Disability Status Scale; RRMS: relapsing-remitting multiple sclerosis; PPMS: primary-progressive multiple sclerosis; RIS: radiologically isolated syndrome; CIS: clinical isolated syndrome.

Bold values indicate a *p*-value ⩽ 0.1.

#### Productivity loss by employment status (Models 1 and 2)

In the model with only unemployed pwMS, women lost an additional 43.0 hours of productivity (95% confidence interval (CI) = −25.4, 111.5) compared to men. In contrast, among employed pwMS, severity was associated with productivity loss. That is, with each one-unit increase in EDSS, we observed an average increase in lost productivity of 18.7 hours (95% CI = 7.1, 30.4). DMT use was only significantly associated with productivity loss among the unemployed, with those on DMTs losing an additional 62.9 hours on average (95% CI = −5.2, 131.0). In addition, on average, employed pwMS who had a relapse within the past 3 months lost an additional 49.2 (95% CI = −12.7, 111.1) hours of productivity relative to those with no relapse.

Among all factors, fatigue was the only indicator consistently significant for the two models using productivity loss as an outcome. Each one-unit increase in the MFIS index (i.e. increasing fatigue) resulted in an average increase in lost productivity of 2.3 (95% CI = 1.6, 3.0) and 5.3 (95% CI = 3.0, 7.6) hours for employed and unemployed pwMS, respectively.

For performance indicators, higher cognitive processing speed and visual acuity scores (signaling improved performance) were associated with less productivity loss for unemployed and employed pwMS, respectively. Specifically, each one-unit increase in the cognitive processing speed and visual acuity indicators resulted in an average decrease in lost productivity of 2.1 (95% CI = −4.9, 0.7) among the unemployed and 0.6 (95% CI = −1.7, 0.5) hours among the employed, respectively.

#### Employment status (Model 3)

As for employment status, for each additional one-unit increase in the EDSS, participants were 1.3 (95% CI = 1.0, 1.7) and 1.5 (95% CI = 1.1, 2.1) times more likely to be unemployed due to health and non-health reasons, respectively, versus being employed full-time. Furthermore, while DMT users compared to non-users were less likely to be unemployed due to non-health reasons (odds ratio (OR) = 0.6; 95% CI = 0.3, 1.1), they were more likely to be unemployed due to health reasons versus employed full-time (OR = 1.8; 95% CI = 1.0, 3.4). Those who experienced a relapse in the last 3 months were more likely to be employed part-time rather than full-time (OR = 2.1; 95% CI = 1.0, 4.4). Likewise, for each additional one-point increase in the MFIS index, participants were more likely to be employed part-time (OR = 1.01; 95% CI = 1.00, 1.03) and unemployed due to health reasons (OR = 1.06; 95% CI = 1.04, 1.08), versus being employed full-time.

Finally, in terms of performance indicators, with each one-unit increase in the cognitive processing speed score (signaling performance improvement), participants were less likely to be employed part-time (OR = 0.95; 95% CI = 0.93, 0.97), unemployed due to non-health reasons (OR = 0.95; 95% CI = 0.93, 0.98), or unemployed due to health reasons (OR = 0.95; 95% CI = 0.92, 0.98), than be employed full-time. Similarly, with each additional one-unit increase in the manual dexterity index (signaling performance deterioration), participants were more likely to be unemployed due to health reasons relative to being employed full-time (OR = 1.03; 95% CI = 1.00, 1.07).

## Discussion

This study is based on comprehensive data of productivity loss among pwMS with early stage disease, using the VOLP, a validated instrument that measures absenteeism, presenteeism, unpaid work productivity loss, and employment status information. Productivity losses were disaggregated by employment status and included MS-related paid work productivity losses (i.e. absenteeism and presenteeism) for those employed, productivity losses incurred by those unemployed (under 65 years old) because of MS (i.e. lost employment time), and unpaid work productivity losses for all. Overall, productivity loss was substantial, reaching 395.4 hours (SD = 309.0) in a 3-month period for the unemployed and 63.6 hours (SD = 107.7) for the employed. Unpaid work loss incurred while doing activities such as childcare or housework among those unemployed surpassed the unpaid work loss of employed pwMS by over three times in a 3-month period. As for employment status, while 75% of the cohort was working at baseline (71% full-time), the other 25% were unemployed, of which 69% reported their unemployment to be related to health issues.

Overall, the unemployment rate of 25% found in this cohort is below the pooled estimate of 35% provided by a recent meta-analysis,^6^ which is likely explained by the lower severity of disability and shorter disease duration for participants in the CanProCo cohort, as well as the regional and temporal differences also outlined in the meta-analysis. As for productivity loss among the employed, results are consistent with those from our previously published study in a CanProCo sub-cohort.^7^ Specifically, the amount and order of importance of each category were similar (presenteeism, followed by absenteeism, and unpaid work). Discrepancies between this study and other comparable studies^11,21^ are likely explained by differences in the instrument used for productivity loss and variation in study subjects, as explained elsewhere.^7^ A comprehensive account, as the one presented here, of productivity losses among all pwMS is missing from the literature. Existing research reports separately on employment status,^6,8^ productivity losses among those working for pay,^11,22^ and costs associated with early retirement and work disability.^4^ The addition and comparison of productivity losses among unemployed pwMS, including unpaid work losses, is an important contribution of this study.

Another key contribution of this study is the exploration of a set of sociodemographic, clinical, MS-related, and performance indicators and their association to the employment-related outcomes of interest. Comparing factors, especially modifiable factors, that are associated with a wide variety of employment outcomes can offer powerful insights in prioritization of policies and resource allocation. Consistent with a literature on determinants of employment status and working hours,^8^ we found fatigue (which in this study accounts for physical, cognitive, and psychosocial dimensions) to be the one factor significantly associated with both employment status and productivity loss indicators. Interestingly, we found that fatigue, which has been identified to be highly prevalent among pwMS,^23^ had a greater impact on productivity losses of unemployed compared to employed pwMS. Considering the low level of disability in our cohort, these findings imply that work-related outcomes (employment status or productivity loss) can be significantly associated with fatigue even before more visible physical disabilities are developed.

Both relapses and overall disability (EDSS) had a significant association with productivity in pwMS who are employed, highlighting the importance of preventing relapses and early disability progression, even at the earliest stages of MS. In contrast, for unemployed pwMS, DMT use in the last 3 months was associated with greater productivity loss. Due to our cross-sectional data and that a substantial proportion of CanProCo participants were newly diagnosed, DMT use could indicate more disease activity beyond other disease severity indicators adjusted in the models. Overall, in light of the high costs of DMTs and their role in driving the economic burden of MS,^24^ early treatment with DMTs has been studied in their capacity to slow progression of disability, prevent relapses, improve QoL, thereby improving productivity and employment-related outcomes.^25,26^ Nevertheless, cost-effectiveness studies shows contradicting results,^27^ and the association of DMTs with productivity loss and employment-related outcomes will need to be further studied over longer periods of time.

This study also incorporates a group of performance indicators for which at least one, either cognitive processing speed, visual acuity, manual dexterity or walking speed, was found to be associated with each of this study outcomes. Specifically, while productivity loss among employed pwMS was associated with visual acuity, productivity loss among unemployed was associated with cognitive processing speed. Furthermore, a deterioration in cognitive processing speed resulted in participants being more likely to be employed part-time or unemployed (regardless of the reason). Furthermore, unemployed pwMS (due to health reasons) were more likely to have worse indicators of manual dexterity. While a previous review identified a variety of performance indicators to be associated with work-related problems in MS,^8^ the inclusion of productivity losses among the unemployed and the variability in effects found across performance indicators shows the importance of individual analyses based on specific work-related outcomes.

One important limitation to note in this study is that the results and conclusions are based on CanProCo participants, which are mostly at an early stage of disease progression and have very mild disease based on EDSS. Consequently, our estimates of productivity loss and unemployed pwMS at baseline are likely an underestimation of the true values at the population level. In other words, we would expect to see higher productivity losses and lower participation in the labor force if the study is expanded to the full MS population in Canada who have more severe disease. In addition, by only using cross-sectional data, we are failing to capture the changes in employment status and productivity loss as the disease progresses. However, in the ongoing longitudinal collection of the CanProCo study, with only 1-year gap between data collection, no substantial changes in the employment and productivity loss indicators of interest are observed (results not presented), and further studies will be conducted after longer follow-up is available. Finally, when calculating lost employment time among the unemployed, we assumed an average number of working hours per week among a general working population in Canada. A more accurate representation would be to include individual employment information of the participant before becoming unemployed (full-time vs part-time employment and hours worked), which is not available at baseline.

This study reinforces the substantial impact that MS has on employment and work productivity, and the importance of consistent and clear disaggregation of productivity loss categories. Making policy and reimbursement decisions in the absence of comprehensive data related to productivity loss could substantially under-estimate the impact of therapy, and ultimately of the cost-effectiveness of new treatments, especially from patient, employer, and societal perspectives. In addition, identifying key factors associated with productivity loss and employment status is key in designing and implementing policies for better workforce retention and improved productivity among pwMS. Such polices could include support for treatments, psychological support, and workplace accommodations including tools to speed up routine tasks such as routine calculations, keeping the work environment cool, minimizing the amount of walking needed, and using tools to offset dexterity issues. This could not only increase QoL by creating the conditions for MS patients to improve their work-related involvement, but it would also lessen the economic burden that MS represents for individuals and for society as a whole.

## Supplemental Material

sj-docx-1-msj-10.1177_13524585231164295 – Supplemental material for Employment status, productivity loss, and associated factors among people with multiple sclerosis Click here for additional data file. Supplemental material, sj-docx-1-msj-10.1177_13524585231164295 for Employment status, productivity loss, and associated factors among people with multiple sclerosis by Elisabet Rodriguez Llorian, Wei Zhang, Amir Khakban, Kristina Michaux, Scott Patten, Anthony Traboulsee, Jiwon Oh, Shannon Kolind, Alexandre Prat, Roger Tam and Larry D Lynd in Multiple Sclerosis Journal
