# Supplementary material for: Employment status, productivity loss, and associated factors among people with multiple sclerosis
Source: Mult Scler. 2023 Apr 15;29(7):866–74. doi: 10.1177/13524585231164295 (PMC10278386; doi:10.1177/13524585231164295)
Supplement: sj-docx-1-msj-10.1177_13524585231164295 – Supplemental material for Employment status, productivity loss, and associated factors among people with multiple sclerosis [file sj-docx-1-msj-10.1177_13524585231164295.docx]

**SUPPLEMENTARY APPENDIX**

**EMPLOYMENT STATUS, PRODUCTIVITY LOSS AND ASSOCIATED FACTORS AMONG PEOPLE WITH MULTIPLE SCLEROSIS**

Elisabet Rodriguez Llorian; Wei Zhang; Amir Khakban; Kristina Michaux; Scott Patten; Anthony Traboulsee; Jiwon Oh; Shannon Kolind; Alexandre Prat; Roger Tam; Larry D Lynd

**Supplementary Methods**

1. **CanProCo Study**

**Inclusion Criteria:** Inclusion criteria for the CanProCo study included being between 18 and 60 years of age, having a confirmed diagnosis of radiologically isolated syndrome (RIS) or relapsing remitting-type of MS (RRMS) within 10 years of disease onset, or primary progressive-MS (PPMS) within 15 years of disease onset, and able to walk at least 100 meters independently or using a walking aid (Expanded Disease Severity Scale (EDSS) under 6.5).

**Ethics approval:** All participating sites obtained local research ethics board approval prior to study initiation.

- Providence St. Joseph’s and St. Michael’s Hospital Healthcare Research Ethics Board, St. Michael’s Hospital (18-325)
- Conjoint Health Research Ethics Board, University of Calgary (18-1853)
- Health Research Ethics Board, University of Edmonton (Pro00086907)
- Clinical Research Ethics Board, University of British Columbia (H18-03047)
- Comité d’éthique de la recherche du Centre hospitalier de l’Université de Montréal (18-293)

**Informed Consent:** Participants must speak and understand English or French to a reasonable degree and be able to independently provide written informed consent to participate in the study. Study related activities are not performed before completion of the informed consent document. Each participating site and funding body has executed required contracts and agreements outlining data access, storage and transfer. All participants have provided consent to publish their de-identified and aggregated data.

**Availability of data and materials:** At completion of the CanProCo study all participating sites will have access to final datasets. After study completion, study data will be made available to qualified external investigators through a data access request and review process.

1. **Productivity Loss**

For absenteeism, participants are asked about the days they were absent from work in the past 3 months because of their health (either due to physical, mental or emotional symptoms). Responses were converted to hours using collected information on participants’ regular working schedules.

Presenteeism was obtained as the percentage of time lost due to health while working, multiplied by the number of days worked (not lost due to absenteeism) in a 3-month period. The percentage of time loss while working follows the formula (a-b)/a, where a = the time taken to complete work in the past 7 days, b =the time that would have taken to complete the same work had the individual experience no health problems.

Lastly, unpaid work time loss in a 3-month period was measured by the hours needed from paid or unpaid help to complete unpaid activities such as housework in the past 7 days multiplied by 13 weeks.

**Supplementary Tables**

**Supplementary Table 1. Factors associated with productivity loss and employment status indicators - Univariate**

|  | **Model 1 - Productivity Losses (hours) Employed PwMS,**  **Marginal Rates** | **Model 2 - Productivity Losses (hours) Unemployed PwMS, Marginal Rates** | **Model 3 - Employment Status, Odds Ratios (Full-time employment is base category)** | | |
| --- | --- | --- | --- | --- | --- |
|  |  |  | **Part-time** | **Unemployed due to health** | **Unemployed not due to health** |
| Female | **0.16 (-20.49, 20.81)** | **28.88 (-50.24, 108.00)** | **2.81 (1.69, 4.68)** | 1.21 (0.82, 1.78) | 1.41 (0.80, 2.49) |
| Age | **0.42 (-0.52, 1.35)** | **5.07 (1.95, 8.18)** | **0.98 (0.96, 0.99)** | **1.04 (1.02, 1.05)** | **0.96 (0.94, 0.99)** |
| EDSS | **23.11 (13.89, 32.32)** | **64.82 (43.88, 85.76)** | 0.96 (0.81, 1.13) | **2.08 (1.79, 2.40)** | 1.17 (0.95, 1.43) |
| Time since diagnosis | **-1.74 (-5.29, 1.81)** | 2.36 (-13.61, 18.33) | 0.99 (0.93, 1.07) | 1.02 (0.95, 1.09) | 1.03 (0.95, 1.13) |
| MS Phenotype |  |  |  |  |  |
| RRMS | 17.51 (-11.56, 46.59) | **65.11 (-261.66, 391.88)** | 1.61 (0.66, 3.91) | **5.12 (1.22, 21.56)** | 1.13 (0.39, 3.30) |
| PPMS | **84.02 (-2.92, 170.97)** | **-48.43 (-244.63, 147.76)** | 0.77 (0.22, 2.74) | **12.92 (2.87, 58.17)** | 1.15 (0.29, 4.64) |
| RIS | -21.81 (-59.38, 15.77) | -162.76 (-367.49, 41.97) | 1.29 (0.42, 3.96) | 1.71 (0.30, 9.91) | 1.50 (0.41, 5.54) |
| CIS | Ref. | Ref. | Ref. | Ref. | Ref. |
| Current DMT use | 12.54 (-5.76, 30.85) | **109.12 (27.85, 190.40)** | 0.96 (0.65, 1.40) | **1.48 (1.03, 2.14)** | **0.58 (0.35, 0.98)** |
| Relapse | **50.92 (-7.61, 109.45)** | -15.84 (-138.00, 106.32) | **2.19 (1.14, 4.18)** | **1.91 (1.02, 3.59)** | **2.06 (0.91, 4.67)** |
| Comorbidities |  |  |  |  |  |
| 0 | Ref. | Ref. | Ref. | Ref. | Ref. |
| 1 | -3.73 (-28.78, 21.32) | -90.63 (-201.32, 20.07) | 0.98 (0.59, 1.62) | 1.41 (0.81, 2.45) | **2.22 (1.23, 4.00)** |
| 2 | 11.85 (-16.67, 40.37) | **50.87 (-56.42, 158.16)** | **1.66 (0.99, 2.78)** | **2.70 (1.56, 4.69)** | 1.02 (0.45, 2.32) |
| 3+ | **57.31 (24.94, 89.69)** | **157.96 (45.76, 270.16)** | 0.98 (0.56, 1.72) | **4.03 (2.45, 6.65)** | 0.59 (0.23, 1.50) |
| Fatigue index MFIS | **2.48 (1.91, 3.04)** | **7.77 (6.36, 9.17)** | **1.02 (1.01, 1.03)** | **1.09 (1.07, 1.10)** | 1.00 (0.99, 1.02) |
| EQ-5D utility score | **-29.90 (-40.98, -18.83)** | **-120.72 (-149.16, -92.27)** | **0.76 (0.63, 0.92)** | **0.42 (0.35, 0.49)** | 1.05 (0.78, 1.43) |
| Cognitive Processing Speed | -0.08 (-0.92, 0.77) | **-4.79 (-8.34, -1.24)** | **0.98 (0.97, 1.00)** | **0.93 (0.91, 0.94)** | **0.96 (0.94, 0.98)** |
| Visual Acuity | **-1.07 (-2.22, 0.08)** | **-20.69 (-38.83, -2.55)** | 1.06 (0.97, 1.16) | **0.97 (0.95, 1.00)** | 1.07 (0.95, 1.21) |
| Manual Dexterity | -0.12 (-1.71, 1.48) | 2.82 (-1.28, 6.91) | 0.98 (0.93, 1.02) | **1.09 (1.06, 1.12)** | **1.07 (1.03, 1.11)** |
| Walking Speed | **9.02 (1.81, 16.23)** | **25.70 (6.86, 44.55)** | 1.06 (0.92, 1.23) | **1.48 (1.32, 1.65)** | **1.29 (1.13, 1.48)** |

Note: bold values indicate a p-value≤0.1. Ref. refers to reference category. Abbreviations: Expanded Disability Disease Scale (EDSS); Relapsing-remitting MS (RRMS); Primary-progressive MS (PPMS); Radiologically isolated syndrome (RIS); Clinical isolated syndrome (CIS).
